# Integrating care for non‐communicable diseases into routine HIV services: key considerations for policy design in sub‐Saharan Africa

**DOI:** 10.1002/jia2.25508

**Published:** 2020-06-19

**Authors:** Alexander Kintu, David Sando, Samson Okello, Gerald Mutungi, David Guwatudde, Nicolas A Menzies, Goodarz Danaei, Stéphane Verguet

**Affiliations:** ^1^ Department of Global Health and Population Harvard T.H. Chan School of Public Health Boston MA USA; ^2^ Department of Internal Medicine Mbarara University of Science and Technology Mbarara Uganda; ^3^ Department of Non‐Communicable Diseases Prevention and Control Ministry of Health Kampala Uganda; ^4^ Department of Epidemiology and Biostatistics School of Public Health College of Health Sciences Makerere University Kampala Uganda; ^5^ Department of Epidemiology Harvard T.H. Chan School of Public Health Boston MA USA

**Keywords:** HIV/AIDS, non‐communicable diseases, antiretroviral therapy, integrated care, sub‐Saharan Africa

## Abstract

**Introduction:**

There is great interest for integrating care for non‐communicable diseases (NCDs) into routine HIV services in sub‐Saharan Africa (SSA) due to the steady rise of the number of people who are ageing with HIV. Suggested health system approaches for intervening on these comorbidities have mostly been normative, with little actionable guidance on implementation, and on the practical, economic and ethical considerations of favouring people living with HIV (PLHIV) versus targeting the general population. We summarize opportunities and challenges related to leveraging HIV treatment platforms to address NCDs among PLHIV. We emphasize key considerations that can guide integrated care in SSA and point to possible interventions for implementation.

**Discussion:**

Integrating care offers an opportunity for effective delivery of NCD services to PLHIV, but may be viewed to unfairly ignore the larger number of NCD cases in the general population. Integration can also help maintain the substantial health and economic benefits that have been achieved by the global HIV/AIDS response. Implementing interventions for integrated care will require assessing the prevalence of common NCDs among PLHIV, which can be achieved via increased screening during routine HIV care. Successful integration will also necessitate earmarking funds for NCD interventions in national budgets.

**Conclusions:**

An expanded agenda for addressing HIV‐NCD comorbidities in SSA may require adding selected NCDs to conditions that are routinely monitored in PLHIV. Attention should be given to mitigating potential tradeoffs in the quality of HIV services that may result from the extra responsibilities borne by HIV health workers. Integrated care will more likely be effective in the context of concurrent health system reforms that address NCDs in the general population, and with synergies with other HIV investments that have been used to strengthen health systems.

## 1 Introduction

The global response to the HIV epidemic in sub‐Saharan Africa (SSA) has resulted in large declines in HIV‐related deaths, and contributed to substantial increments in life expectancy [1]. Despite this progress, new adult infections remain high in the region with over 900,000 new cases reported in 2018 [2]. Additionally, for many countries, the epidemic is becoming more concentrated in middle‐age groups that also have a large proportion of individuals who are at high risk for non‐communicable diseases (NCDs) [3]. For example, in Uganda, although the prevalence of HIV had reduced to 6% by 2017, 14% of men and 11% of women aged 45 to 49 years were HIV positive [4].

With the rapidly increasing number of people who are ageing with HIV and the resulting steady rise of NCDs in this population [5], there has been growing enthusiasm and advocacy for integrating care for NCDs into routine HIV treatment services in SSA [6]. In spite of a similar increase in the prevalence of NCDs in the general population, interventions for NCDs are rarely included in national primary care packages and are often paid for via out‐of‐pocket medical payments, which can lead to “catastrophic” expenditure and medical impoverishment [7]. Also, the global AIDS response that is primarily donor‐supported has been set up to address HIV and common opportunistic infections, with limited focus on other conditions [8]. This has resulted in significant gaps in the cascade of care for NCDs in countries that are on track to meeting the aspirational 90‐90‐90 targets [9].

In SSA, integration of care for other conditions with HIV services at a national level has perhaps been best demonstrated for prevention of mother‐to‐child transmission of HIV with 85% coverage achieved [10]. Capitalizing on the remarkable achievements and lessons learnt from the HIV/AIDS response, integrating services for HIV and NCDs could enable the delivery of appropriate, and affordable healthcare to millions in need [11]. The narrative on integrated care for HIV and NCDs in SSA has mostly emerged from the perspective of improving access to interventions for NCD services for people living with HIV (PLHIV) [6, 11]. Less attention has been devoted to the in‐depth examination of the practical, economical and ethical considerations of prioritizing PLHIV as opposed to targeting the general population that is also experiencing a rapidly growing prevalence of NCDs. Furthermore, suggested health system approaches for addressing HIV‐NCD comorbidities have mostly been normative, and have provided little actionable guidance on how to implement specific interventions, especially in SSA [12, 13]. We summarize key opportunities and challenges for using HIV treatment platforms to address NCDs among PLHIV. We emphasize essential considerations that can guide integrated care in SSA and point to possible interventions for implementation.

## 2 Discussion

The increasing rates of HIV‐NCD comorbidities present significant challenges to health systems in many low‐ and middle‐income countries (LMICs), that are technically and financially constrained, and have mostly been designed to respond to communicable, maternal and childhood conditions. Policymakers in these countries are now faced with a decision on whether to favour interventions for integrating NCD care and treatment into existing HIV services, as opposed to embarking on broader health system reforms for addressing NCDs in the general population. There are substantially more NCD cases among HIV‐negative individuals essentially because of their larger proportion within the total population when compared to PLHIV. Proponents of broader reforms may support the principle of using a “veil of ignorance” and thereby not considering individuals’ characteristics (in this case HIV‐NCD comorbidities) when allocating scarce resources [14]. Focusing on the general population is also a more equitable option, but would inevitably be costlier and more difficult to implement. In contrast, an approach that favours integrating care for NCDs with existing HIV services would potentially be easier and more affordable to implement because of the regular visits that PLHIV make to health facilities. The already existing counselling and laboratory services, skilled human resources and drug supply chain mechanisms of ART delivery could be expanded and utilized to deliver high‐quality services for NCD care to PLHIV with more manageable added costs. Integrating HIV and NCD care could also potentially lead to economies of scale (i.e. decrease in costs as programmes expand in volume) and economies of scope (i.e. decrease in costs as programmes jointly provide multiple services onto the same delivery platform), and thus improve efficiency, patient outcomes and enhanced responsiveness to local needs [15]. Choosing to first address NCDs in PLHIV might however be seen as unfair, as it would ignore the larger number of NCD cases in the general population [16].

We propose that countries embark on gradual integration of NCD care into existing HIV services, and view this as a means of providing the necessary comprehensive care to people who are ageing with comorbidities. We acknowledge that this approach may increase the current disparities in access to healthcare between PLHIV and the general population. Yet, inaction could otherwise jeopardize the substantial health and economic benefits that have been achieved while scaling up ART services. We further propose that this integration be implemented concurrently with broader health system reforms that address NCDs in the general population, which would in part help mitigate the inequalities created by prioritizing PLHIV.

A second key consideration for integrated care is identifying specific conditions to prioritize in HIV treatment programmes. The extensive literature documenting the increasing rates of HIV‐NCD comorbidities is mostly from subnational studies that have reported on conditions like obesity and hypertension [17, 18, 19, 20, 21]. Some studies have also provided pooled estimates on the prevalence of these comorbidities in LMICs [5]. However, country‐specific estimates on the prevalence of common NCDs in PLHIV remain largely unknown for most of SSA. HIV treatment programmes generally do not track NCD comorbidities essentially because there are no associated reporting requirements for these conditions. Most countries also do not have national surveys that jointly collect data on HIV status and NCDs. The World Health Organization‐supported STEPS surveys on NCD risk factors that are available in many LMICs do not collect information on HIV status [22]. Likewise, the Population‐based HIV Impact Assessments that are now widely used for estimating HIV prevalence in SSA only focus on HIV [3]. Indeed, more granular information on the prevalence of HIV‐NCD comorbidities would be required to determine the cost of integrated care and set priorities. For example, it was recently modelled that in Uganda it might be more cost‐effective to target older PLHIV (45 years or older) for routine screening for hypertension, diabetes and dyslipidemias because of the higher prevalence of the three conditions at these ages [23].

To address some of the current information gaps, we propose that HIV treatment programmes prioritize routine screening of NCDs that are commonly seen in PLHIV (Box 1). These conditions can also be added to the set of indicators that are routinely monitored by ART delivery programmes. Specific attention could be given to regular body weight assessments because of the increasing rates of excessive weight gain among patients on ART (Table 1) [19, 21, 24]. Excessive weight gain is concerning because a high body mass index (BMI) is a major risk factor for several NCDs and has been linked to increased risk for all‐cause mortality [25]. Body weight assessments are already part of routine HIV care but current guidance mostly focuses on weight gain as a positive sign of immune recovery. Emphasis can also be put on improving the capacity of health facilities to measure height and on periodic interpretation of BMI values. Similarly, more routine blood pressure assessments could be undertaken because of the observed high rates of hypertension in PLHIV [19, 21]. Enhanced blood pressure screening would also help address the gaps in the cascade of care for hypertension that have been observed in HIV treatment programmes with high levels of virologic suppression [9].

**Table 1:** Non‐communicable disease risk factors and conditions for consideration for screening among people living with HIV in sub‐Saharan Africa

Risk factor/disease	Prevalence (95% CI)	Proposed intervention
Smoking	Men: 25.9% (24.6 to 27.3) Women: 1.2% (0.9 to 1.4) [32]	Increase screening and counselling on smoking and tobacco use
Weight gain and obesity	27.3% (20.2 to 35.9) [5]	Implement recommended body weight monitoring in routine HIV care Carry out periodic tracking of proportion of overweight or obese PLHIV in HIV treatment programmes
Hypertension	21.2% (16.3 to 27.1) [5]	Increase blood pressure monitoring in routine HIV care Carry out periodic tracking of proportion of hypertensive PLHIV in HIV treatment programmes
Hypercholesterolemia	22.2% (14.7 to 32.1) [5]	Prioritize determining the prevalence of dyslipidemias in PLHIV to identify high‐risk age groups
Diabetes mellitus	1.3 to 18.0%^a^ [5]	Prioritize determining the prevalence of diabetes mellitus in PLHIV to identify high‐risk age‐groups

BMI, body mass index; PLHIV, People living with HIV.

a Estimates only available as a range.

Box 1 **Policy implications**

We recommend the following to be considered in addressing the growing prevalence of NCDs in people who are ageing with HIV in sub‐Saharan Africa.

- There is an urgent need for expanding the global agenda for combating the HIV epidemic to include care for common NCDs in PLHIV, so to sustain the substantial health and economic benefits that have been achieved with scaling up ART services.
- Integrating NCD care into existing HIV services should be viewed as a means of providing the necessary comprehensive care to PLHIV ageing with comorbidities.
- Broader health system reforms to address NCDs in the general population will be essential to improve access to services for conditions that might not be adequately managed in HIV treatment settings.
- Prevalence and incidence of common NCDs in PLHIV can be included in the set of indicators that are routinely monitored by ART delivery programmes.

These suggested intensified screening procedures would be implemented while taking into consideration the possibility of further straining an already burdened health system. Although integrated care can lead to better patient outcomes [15], additional responsibilities on health workers could compromise the quality of existing HIV services. As a first step, changes in current practice can be limited to a few services, and preferably those that are already part of recommended care and use existing infrastructure. For example, although HIV programmes can be used for cervical cancer screening and management, the feasibility of such a programme might be compromised by the high costs incurred [26]. Cervical cancer programmes may therefore be more attainable as part of a nationwide strategy targeting the general population.

At the national level, periodic surveys could be designed to collect information on both HIV/ART status and common NCDs and their risk factors. The STEPS surveys could be modified to collect information on HIV status. This modification would also provide required estimates on the prevalence of conditions that are difficult to assess in routine HIV care, such as diabetes and dyslipidaemias.

Lastly, successful implementation of integrated care will require mobilizing additional funds to expand HIV treatment programmes to intensified screening and management of NCDs. In spite of the increasing prevalence of NCDs in SSA, current funding for NCD interventions mostly comes from domestic sources and has been shown to be inadequate [27, 28]. Many countries now have strategic plans for addressing NCDs but implementation of the identified interventions has been limited by severe financial constraints [29]. In addition, although medications for common NCDs are now included in most national Essential Medicines Lists, frequent stockouts and inadequate management of drug supply chains repeatedly occur at health facilities in the public sector [30]. These funding and health system constraints have contributed to gaps in access to NCD services for PLHIV [9]. Similar gaps have been documented in the general population which has fewer encounters (compared with PLHIV) with the health system [31].

Addressing these substantial financing challenges will necessitate mobilizing additional funds for delivering high‐yield NCD services to PLHIV and the general population. As global agencies recognize the risks posed by the rise of NCDs among ageing PLHIV, there is a need for coordinated efforts to address HIV‐NCD comorbidities. However, given that global funding for HIV/AIDS programmes has plateaued in recent years [11], the interventions that we propose for consideration are more likely to succeed in the context of increased budgetary allocation to NCDs by ministries of health in the short term. Increased domestic allocations may also in part reduce the inequalities that would be created by interventions that would only target PLHIV. There is also a need for more research on how to capitalize on the remarkable achievements of the HIV/AIDS response to deliver high‐yield services to the general population.

## 3 Conclusions

An expanded agenda for addressing common NCDs in PLHIV can help maintain the substantial health and economic benefits that have been achieved by the HIV/AIDS response.

Integrated care for NCDs in HIV treatment settings will require country‐specific estimates on the proportion of PLHIV who need access to NCD services. Successful integration will also require earmarking additional funding for delivering high‐yield NCD services for PLHIV. Attention should be given to mitigating potential tradeoffs in the quality of HIV services that may result from the extra responsibilities borne by HIV health workers, and to evaluating ways of scaling up successful interventions in HIV treatment settings to the general population.

Although integrated care can increase access to necessary services, the interventions we suggest are more likely to be effective in the context of gradual health system reforms for addressing NCDs in the general population. Investments in broader reforms will be essential to improve access to services for conditions that might not be adequately provided for in HIV treatment settings, such as cancer screening and treatment, as well as long‐term care of mental health disorders in PLHIV. Advancing care for these conditions will require improvements in patient referral mechanisms, better use of Health Management Information Systems, and increasing access to specialized services. Likewise, synergies with other existing health investments should be prioritized, such as the HIV investments that have been used to strengthen health systems.

## Competing interest

We declare no competing interests.

## Authors’ Contributions

AK conceived the policy suggestions that are discussed in this commentary in consultation with all co‐authors. AK wrote the first draft of the manuscript, under supervision of SV. DS, DG, SO, GM, NAM and GD provided advice on the content of the manuscript. All authors contributed to writing and reviewing the manuscript.
